# Association of Fear and Anxiety Scales in Pediatric Dental Patients Using Brainwave Entrainment: A Randomized Controlled Trial

**DOI:** 10.7759/cureus.66526

**Published:** 2024-08-09

**Authors:** Farah Shehani Areef, Victor Samuel Andiyappan, Kavitha Ramar

**Affiliations:** 1 Pediatric and Preventive Dentistry, Sri Ramaswamy Memorial (SRM) Kattankulathur Dental College and Hospital, SRM Institute of Science and Technology, Kattankulathur, IND

**Keywords:** spearman's correlation, binaural beats, non-pharmacological interventions, alpha wave entrainment, pediatric dentistry, dental anxiety, dental fear, brainwave entrainment, behavioral scales, randomized controlled trial

## Abstract

Background

Dental fear and anxiety are significant issues among pediatric patients, often complicating dental treatments. Various tools measure these emotional responses, including subjective scales such as the Visual Facial Anxiety Scale (VFAS) and Children Fear Scale (CFS), and objective scales such as Venham’s Anxiety Scale (VAS) and Frankl Behavior Rating Scale (FBRS). This study explores the association between these measures in children subjected to brainwave entrainment (BWE) therapy. This study aimed to evaluate the association between subjective and objective fear and anxiety measures in pediatric dental patients within both the brainwave entrainment (BWE) intervention group and control group.

Methods

This randomized controlled trial included pediatric participants aged seven to 12 years reporting to the department for dental treatment. Participants were randomized into two following groups: an experimental group receiving BWE therapy and a control group receiving traditional behavioral management. Fear and anxiety levels were measured using subjective and objective scales before and after the intervention. Data were analyzed using Spearman’s correlation to examine the associations between these scales, with statistical significance set at p<0.05.

Results

Post-intervention analysis revealed significant correlations between subjective and objective measures of fear and anxiety in both groups. In the BWE group (N=126), there was a moderate positive correlation between the VFAS and VAS (rho=0.540, p<0.001) and a strong negative correlation between the CFS and FBRS (rho=-0.666, p<0.001). The control group (N=126) showed stronger correlations, rho=0.778 for anxiety scales and rho=-0.817 for fear and behavior scales (p<0.001). Combined data analysis from both groups (N=252) confirmed strong correlations.

Conclusion

This study found a significant association between subjective and objective measures of fear and anxiety in pediatric dental patients within both the brainwave entrainment (BWE) intervention group and control group. Thereby proving that self-reporting behavioral scales are useful for quickly assessing anxiety in pediatric dental settings.

## Introduction

Dental fear and anxiety are prevalent issues among pediatric patients, often leading to uncooperative behavior that complicates dental treatment [1,2]. These emotional responses can significantly impact the child’s dental health and overall well-being, making it essential to identify and manage dental anxiety effectively [3,4].

Various tools have been developed to measure dental anxiety and fear in children. Among the most widely used are the Children's Fear Survey Schedule-Dental Subscale (CFSS-DS) and the Modified Child Dental Anxiety Scale (MCDAS) [5,6]. These tools have been validated in different languages and cultural contexts, demonstrating their reliability and utility in clinical settings [7,8]. However, there is an ongoing need to explore the correlation between subjective and objective measures of dental anxiety to enhance the assessment and intervention strategies.

Subjective measures, such as self-reported anxiety scales, provide direct insight into the patient’s perceived anxiety and fear [9]. These scales are easy to administer and interpret, making them practical for use in busy clinical settings. On the other hand, objective measures include behavioral assessments conducted by clinicians, such as Venham’s Anxiety Scale (VAS) and the Frankl Behavior Rating Scale (FBRS) [10,11]. These scales offer a more detached evaluation of the child’s anxiety and fear, potentially providing a more objective perspective on the patient’s emotional state.

Research has shown that subjective measures can often reflect objective outcomes. For instance, Armfield (2010) [4] and Howard and Freeman (2007) found that children’s self-reported anxiety levels were significantly correlated with physiological and behavioral indicators of distress [12]. This suggests that subjective measures, despite their inherent biases, can be reliable indicators of dental anxiety and fear.

The use of brainwave entrainment (BWE) therapy as an intervention for reducing dental anxiety in children has gained attention in recent years. BWE therapy involves the use of rhythmic auditory stimuli to synchronize brainwave frequencies, potentially inducing a state of relaxation [13]. However, the effectiveness of BWE therapy in reducing dental anxiety and its impact on the correlation between subjective and objective measures remains underexplored.

To our knowledge, this study is novel in its approach to evaluating the correlation between subjective and objective fear and anxiety measures in pediatric dental patients subjected to brainwave entrainment intervention. This untapped aspect of research highlights the innovative nature of our investigation.

Therefore, this study aimed to evaluate the correlation between subjective and objective fear and anxiety measures in pediatric dental patients within both the brainwave entrainment (BWE) intervention group and the control group. Understanding the relationship between these measures can enhance assessing and managing dental anxiety and fear in pediatric dental patients, ultimately improving patient outcomes and clinical practices.

## Materials and methods

Study design

**Figure 1:**
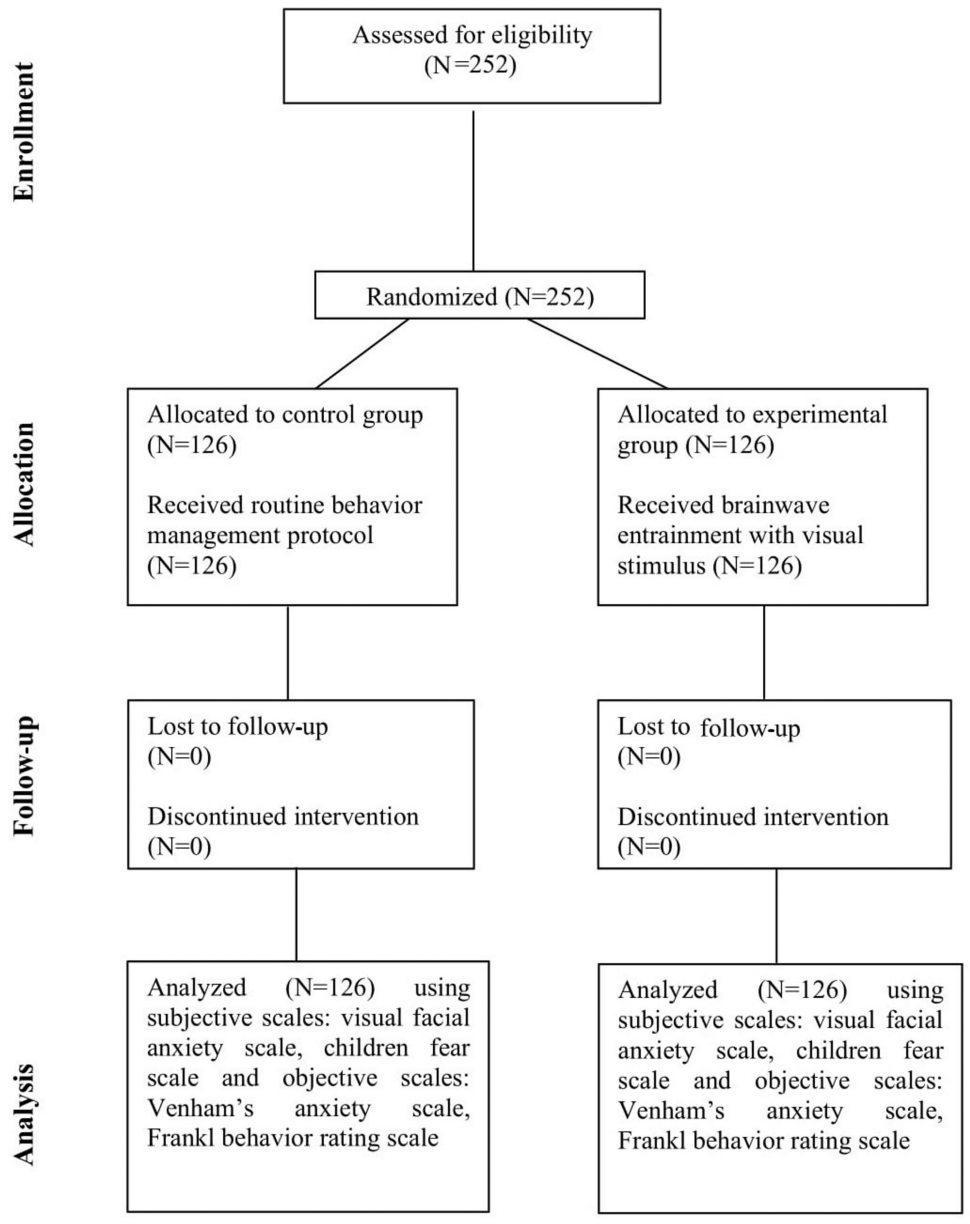
CONSORT flow chart detailing participant recruitment, allocation, follow-up, and analysis. CONSORT: Consolidated Standards of Reporting Trials N: number of participants

This randomized controlled trial aimed to assess the relationship between subjective and objective anxiety and fear measures in pediatric dental patients. The study compared outcomes between a brainwave entrainment (BWE) group and a control group. The Institutional Ethical Committee approved the study protocol (reference number 3010/IEC/2021), and it was registered in the Clinical Trial Registry of India (CTRI) with the reference code CTRI/2023/03/051066. The study data detailing participant recruitment, allocation, follow-up, and analysis is represented in the Consolidated Standards of Reporting Trials (CONSORT) flow chart (Figure 1).

Sample size estimation

The required sample size for our study was calculated using G*Power software (Düsseldorf, Germany: Heinrich Heine University) with specific parameters to ensure statistical robustness and sensitivity. The parameters included an expected effect size (Cohen's d) of 0.5, a significance level (α) of 0.05, and a desired statistical power (1-β) of 0.95. These criteria determined a necessary sample size of 252 participants.

Participants

Inclusion Parameters

The study focused on participants aged seven to 12 years selected based on their cooperative behavior as indicated by positive Frankl behavior scale evaluations. Enrollment was limited to children subjected to dental treatments within the pediatric dental department.

Exclusion Parameters

Participants were excluded if they exhibited uncooperative behaviors requiring additional behavioral management during dental procedures. Additionally, children with pre-existing medical conditions that could be affected by brainwave entrainment, such as neurological disorders, brain injuries, or sensory impairments, were not included. Refusal of consent by either the children or their legal guardians also resulted in exclusion from the study.

Randomization protocol

The study utilized the digital tool "randomizer.org" to create 126 unique numerical identifier pairs ranging from 1 to 2. To uphold methodological integrity and ensure blinding, participant details were enclosed in opaque, sequentially numbered envelopes. This process enabled the fair random allocation of 252 subjects into two following groups: an experimental group and a control group. The group assignments were kept concealed from the data analyst until the study was completed.

Informed consent process

The study's methodology was comprehensively communicated to both the participants and their guardians or parents, including a detailed description of the audiovisual (AV) stimulation to be employed. Written consent was secured from the guardians or parents, while the participants provided verbal assent.

Intervention methodology

The experimental group received a binaural beat stimulus aimed at inducing alpha wave (10 Hz) entrainment through differential auditory inputs (left stimulus: 9.5 Hz, right stimulus: 10.5 Hz) administered using the "DAVID Delight Plus Device Software" (Alberta, Canada: Mind Alive Inc.). This setup included compatible headsets and a multicolored eyeset for smartphone connectivity [14]. Conversely, the control group underwent traditional behavioral management techniques, including tell-show-do, tell-play-do, euphemisms, modeling, and distraction. Each intervention lasted 10 minutes. The volume was standardized at a sound pressure level of 60 dB, with adjustments allowed for participant comfort. To minimize external auditory disturbances, ambient clinical noise was reduced, and participants were instructed to keep their eyes closed throughout the intervention. To prevent bias, the principal investigator's interactions with participants were limited to essential procedural instructions.

Assessment measures

Subjective scales included the Visual Facial Anxiety Scale (VFAS), which assessed anxiety based on facial expressions interpreted by the dental practitioner, and the Children Fear Scale (CFS), a self-reported measure where children indicated their level of fear related to the dental environment on a pictorial scale. Objective scales comprised the Venham’s Anxiety Scale (VAS), used by an observer to rate the child’s anxiety based on behavioral cues during the dental visit, and the Frankl Behavior Rating Scale (FBRS), which evaluated the child's cooperative behavior during dental treatment, ranging from definitely negative to definitely positive behavior.

Data collection

Data were collected immediately before and after the intervention in both the control and intervention groups. Anxiety and fear levels were measured using the subjective and objective scales mentioned above.

Statistical analysis

Data were analyzed using Spearman’s correlation to examine the associations between the subjective and objective measures of anxiety and fear. Comparisons between pre- and post-intervention measurements within and between groups were conducted using paired and unpaired t-tests. A p-value of less than 0.05 was considered statistically significant.

## Results

The demographic characteristics of the sample of 252 participants were presented in Table 1 as follows: the age distribution revealed that the largest group consisted of seven-year-olds, with 95 participants, making up 37.7% of the sample. This was followed by nine-year-olds, who accounted for 18.3% (46 participants), and 10-year-olds, who represented 13.9% (35 participants). The sample also included 33 participants aged 12 years (13.1%), 24 participants aged eight years (9.5%), and 19 participants aged 11 years (7.5%). In terms of gender, the sample was slightly male-dominated, with 134 male participants (53.2%) compared to 118 female participants (46.8%). Overall, the sample was characterized by a higher number of younger children, particularly those aged seven, and a nearly balanced gender distribution.

**Table 1:** The demographic characteristics of the study participants. N: number of participants

Demographic characteristics	Frequency (N)	Percent (%)
Age (years)		
7	95	37.7
8	24	9.5
9	46	18.3
10	35	13.9
11	19	7.5
12	33	13.1
Gender		
Male	134	53.2
Female	118	46.8

Data distribution and implications

The normality of the data was assessed using the Shapiro-Wilk test, which indicated a non-normal distribution (p<0.05). As a result, Spearman's rank-order correlation was utilized to analyze the relationships between subjective and objective measures. This non-parametric method is suitable for ordinal data or when normality assumptions are not met, offering a robust analysis of correlations. This choice ensures the reliability of the findings in the context of non-normally distributed data.

Spearman's correlation analysis as seen in Table 2 was utilized to assess the relationship between various scales measuring anxiety and behavior post-intervention in different groups. This non-parametric test evaluates the strength and direction of the association between two variables, using a monotonic function. In this study, it was applied to understand the relationship between objective measures (such as heart rate and physiological data) and subjective assessments (like self-reported anxiety and behavioral observations) following the intervention. This approach provided insights into how these different measures of anxiety and behavior correlated after the study's interventions.

**Table 2:** Association between subjective and objective anxiety and fear scales. *Spearman's correlation coefficient test was used, with results considered statistically significant at the p=0.01 level (2-tailed). N: number of participants; EXP: brainwave entrainment group (N=126); CON: control group (N=126); overall: combined group (N=252)

Group	Subjective scale	Objective scale	p-Value
EXP group (N=126)	Visual Facial Anxiety Scale	Venham’s Anxiety Scale	0.540 (0.000)*
EXP group (N=126)	Children Fear Scale	Frankl Behavior Rating Scale	-0.666 (0.000)*
CON group (N=126)	Visual Facial Anxiety Scale	Venham’s Anxiety Scale	0.778 (0.000)*
CON group (N=126)	Children Fear Scale	Frankl Behavior Rating Scale	-0.817 (0.000)*
Overall (N=252)	Visual Facial Anxiety Scale	Venham’s Anxiety Scale	0.867 (0.000)*
Overall (N=252)	Children Fear Scale	Frankl Behavior Rating Scale	-0.920 (0.000)*

Correlation statistics

The results of Spearman's correlation analyses in this study, as shown in Table 2, examine the relationships between subjective and objective measures of anxiety and fear in both the brainwave entrainment (BWE) and control groups, involving 252 participants.

BWE Group (N=126)

Post-intervention analysis revealed a moderate positive correlation between the Visual Facial Anxiety Scale and Venham’s Anxiety Scale (rho=0.540, p<0.001), indicating consistent anxiety reporting across measures. A strong negative correlation was observed between the Children Fear Scale and the Frankl Behavior Rating Scale (rho=-0.666, p<0.001), suggesting that higher fear scores correlate with worse behavior.

Control Group (N=126)

Similar but stronger correlations were found, with rho=0.778 for anxiety scales and rho=-0.817 for fear and behavior scales (both p<0.001), indicating more pronounced effects or clearer data patterns due to the absence of the intervention.

Combined Data (N=252)

The combined analysis highlighted strong correlations, with a positive rho=0.867 for the anxiety scales and a negative rho=-0.920 for fear and behavior scales (both p<0.001), reinforcing the reliability and consistency of these measures in assessing pediatric dental anxiety and behavior. This data underscores the importance of these scales in clinical evaluations and highlights the distinct impact of BWE intervention in managing anxiety and behavior in pediatric dental patients.

## Discussion

Dental anxiety, especially among children, poses a major challenge in dental care, requiring solutions that go beyond traditional medications [15]. The findings of this study highlight the significant correlations between subjective and objective anxiety and fear scales in pediatric dental patients, which have several important implications for both clinical practice and future research. The study found that subjective measures such as the Visual Facial Anxiety Scale (VFAS) and the Children Fear Scale (CFS) strongly correlate with objective measures such as Venham’s Anxiety Scale (VAS) and the Frankle Behavior Rating Scale (FBRS). This indicates that subjective measures can reliably reflect objective outcomes, which is crucial for developing effective strategies for managing dental anxiety and fear in children.

Firstly, the significant correlations observed suggest that subjective measures can be effectively used for initial assessments and ongoing monitoring of anxiety and fear in pediatric dental patients. This is particularly valuable in clinical settings where quick and non-invasive assessment tools are needed. Subjective scales are easy to administer and interpret, making them practical for use in busy dental practices. The VFAS, developed to provide a user-friendly and quick assessment of anxiety, allows children to express their level of fear straightforwardly, providing immediate feedback to the clinician. This can help in tailoring the dental experience to the child's comfort level, thereby reducing anxiety and improving cooperation during dental procedures [5,9].

Secondly, the differences in correlation strength between the BWE group and the control group provide insight into the effectiveness of brainwave entrainment (BWE) therapy. The control group exhibited higher correlations between subjective and objective measures compared to the BWE group. This suggests that while both subjective and objective measures are reliable, the BWE therapy may influence how these measures correlate. BWE therapy may help in reducing the overall anxiety and fear levels, thus affecting the strength of the correlation. This indicates a potential moderating effect of BWE therapy on the relationship between subjective and objective measures, which warrants further investigation [13].

Moreover, the strong correlations found in the overall analysis (combining both groups) underscore the robustness of the subjective measures in predicting objective outcomes. The correlation of 0.867 between the Visual Facial Anxiety Scale (VFAS) and Venham’s Anxiety Scale (VAS), and -0.920 between the Children Fear Scale (CFS) and Frankle Behavior Rating Scale (FBRS), demonstrates that subjective measures are not only reflective of objective states but also consistent across different groups and conditions. The positive correlations confirm that subjective and objective anxiety measures are consistent, while the negative correlations emphasize how increased fear can negatively impact cooperative behavior. This consistency is crucial for validating the use of these scales in various clinical settings and populations [9].

In this study, only post-intervention assessments were conducted and reported because the pre-assessment values for all participants were uniformly zero. This was done to ensure standardization and a consistent baseline for measuring the effectiveness of brainwave entrainment (BWE) therapy. By selecting cooperative participants, we were able to focus on assessing the specific effects of BWE therapy on dental anxiety and fear without confounding factors related to initial uncooperativeness. This approach allowed us to evaluate the potential of BWE therapy in a controlled and systematic manner. This outcome was anticipated since the study exclusively included cooperative children, who inherently displayed no significant baseline anxiety or fear. As a result, the pre-assessment measurements lacked meaningful variability and did not provide useful data for analysis. By focusing on post-intervention assessments, we aimed to accurately capture the changes induced by the interventions, facilitating a more precise evaluation of the effects of both brainwave entrainment (BWE) therapy and conventional behavioral management techniques on pediatric dental anxiety and fear.

The study's findings are consistent with previous research indicating that subjective measures can reflect objective outcomes in clinical settings. For instance, studies have shown that children’s self-reported anxiety levels often correlate with physiological and behavioral indicators of distress [12,16]. This alignment between subjective and objective measures validates the use of self-reported scales for assessing dental fear and anxiety. Additionally, the results align with the broader psychological literature which supports the use of subjective measures in clinical assessments. For example, the use of self-reported scales in assessing anxiety, depression, and other psychological conditions has been well-documented and validated [3].

One of the major implications of this study is the potential for subjective measures to enhance the feasibility and efficiency of dental anxiety assessments. Given their ease of use and non-invasiveness, subjective scales can be quickly administered in clinical settings, allowing for rapid assessment and intervention. This is particularly beneficial in pediatric dentistry, where children’s cooperation and comfort are paramount. By using subjective measures, clinicians can gain immediate insights into a child’s emotional state and adjust their approach accordingly. This can lead to better management of dental anxiety, improved patient experiences, and ultimately, better dental health outcomes [2].

Furthermore, the study highlights the need for further research into the mechanisms underlying the observed correlations. Understanding why and how subjective measures correlate with objective outcomes can provide deeper insights into the nature of dental anxiety and fear. For example, exploring factors such as the child’s previous dental experiences, parental influence, and individual psychological traits could help in refining the assessment tools and developing more targeted interventions. Additionally, investigating the role of BWE therapy in moderating these correlations can provide valuable information on its efficacy and potential applications in pediatric dentistry [17].

The findings of this study also have practical implications for dental practitioners. By incorporating subjective measures into routine assessments, practitioners can better identify children at risk of high anxiety and fear, allowing for timely interventions. For example, children identified as highly anxious can be provided with additional support, such as desensitization sessions, behavioral therapy, or sedation options. This proactive approach can help reduce the overall anxiety levels and improve the dental experience for pediatric patients [18].

Despite the strengths of the study, certain limitations need to be addressed. The generalizability of the findings may vary with region-specific demographic characteristics [19-20]. Another limitation is the potential for response bias in subjective measures. While subjective scales are valuable for capturing the child’s self-reported anxiety and fear, they can be influenced by factors such as social desirability and the child’s current mood. Furthermore, the study did not explore the potential differences in anxiety and fear levels across different types of dental procedures. Certain procedures, such as extractions or fillings, may induce higher levels of anxiety compared to routine check-ups or cleanings. The registered Clinical Trials Registry of India (CTRI) includes essential parameters, in addition to the four psychometric scales referenced here, for assessing dental fear and anxiety in pediatric patients. However, in the present study, we have confined our analysis to the comparison of these four scales.

## Conclusions

In conclusion, this study revealed a significant correlation between subjective and objective fear and anxiety measures in pediatric dental patients within both the brainwave entrainment (BWE) intervention and control groups. These findings indicate that self-reported measures of anxiety can be effectively used for quick and practical assessment in clinical settings, complementing objective measures. The effective reduction of anxiety levels by BWE therapy, as indicated by the correlation data, suggests its potential as a non-pharmacological intervention for managing dental anxiety in pediatric patients. Future research should continue to explore the mechanisms behind these correlations and the potential moderating effects of BWE therapy to further enhance pediatric dental care.
